# Critical Importance of Long-Term Adherence to Care in HIV Infected Patients in the cART Era: New Insights from *Pneumocystis jirovecii* Pneumonia Cases over 2004–2011 in the FHDH-ANRS CO4 Cohort

**DOI:** 10.1371/journal.pone.0094183

**Published:** 2014-04-11

**Authors:** Blandine Denis, Marguerite Guiguet, Nathalie de Castro, Frédéric Mechaï, Matthieu Revest, Aba Mahamat, Giovanna Melica Gregoire, Olivier Lortholary, Dominique Costagliola

**Affiliations:** 1 Inserm UMRS 1136, Paris, France; 2 UPMC Univ Paris 06 UMRS 1136, Paris, France; 3 Service de maladies infectieuses et tropicales, CHU Saint Louis, AP-HP, Paris, France; 4 Université Paris Diderot, Paris, France; 5 Service de maladies infectieuses et tropicales, CHU Avicenne AP-HP, Bobigny, France; 6 Service de maladies infectieuses et tropicales, CHU Rennes, Rennes, France; 7 Service de maladies infectieuses et tropicales, CHU Cayenne, Cayenne, France; 8 EA 3595, Univ Antilles-Guyane, Cayenne, France; 9 CHU Necker-Enfants malades, AP-HP, Centre d'Infectiologie Necker Pasteur, Paris, France; 10 Université Paris Descartes, Institut Imagine, Paris, France; 11 Institut Pasteur, Centre National de Référence Mycoses Invasives et Antifongiques, Paris, France; 12 CNRS URA 3012, Paris, France; Temple University School of Medicine, United States of America

## Abstract

**Objective:**

To describe characteristics and outcomes of HIV-infected patients with *Pneumocystis jirovecii* pneumonia (PCP) over 2004–2011 in France, in particular in those previously enrolled (PE) in the French Hospital Database on HIV (FHDH).

**Methods:**

PE patients with an incident PCP were compared with patients with an inaugural PCP revealing HIV infection (reference). Adequate adherence to care was defined as a CD4 measurement at least every 6 months. Immune reconstitution (CD4≥200/mm^3^) and risk of death were studied using Kaplan-Meier estimates and multivariable Cox proportional hazards models.

**Results:**

In a context of a decreasing incidence of PCP, 1259 HIV-infected patients had a PCP diagnosis, and 593 (47%) were PE patients of whom 161 (27%) have had a prior history of AIDS-defining clinical illness (prior ADI). Median time since enrolment was 8 years for PE patients; 74% had received cART. Median proportion of time with adequate adherence to care was 85% (IQR, 66–96) for all FHDH enrollees, but only 45% (IQR, 1–81) for PE patients during the 2 years before PCP. Median CD4 cell count (38/mm^3^) and HIV viral load (5.2 log10 copies/ml) at PCP diagnosis did not differ between PE patients and the reference group. Three year mortality rate of 25% was observed for PE prior ADI group, higher than in PE non-prior ADI group (8%) and the reference group (9%) (p<0.0001). In the PE prior ADI group, poor prognosis remained even after adjustment for virological control and immune reconstitution (HR, 2.4 [95%CI, 1.5–3.7]).

**Conclusion:**

Almost 50% of PCP diagnoses in HIV-infected patients occurred presently in patients already in care, mainly with a previous cART prescription but with waning adherence to care. Having repeated ADI is contributing to the risk of death beyond its impact on immune reconstitution and viral suppression: special efforts must be undertaken to maintain those patients in care.

## Introduction

*Pneumocystis jirovecii* pneumonia (PCP) is a well-recognized major opportunistic infection and one of the most common AIDS-defining illnesses (ADI) [1]–[3]. Before the introduction of Pneumocystis prophylaxis and combination antiretroviral therapy (cART), it was estimated that 75% of HIV infected patients developed PCP during their lifetime [3], [4]. After 1996, with the availability of cART, a dramatic decline in PCP incidence was observed in HIV infected patients [5]. In previous studies, the proportion of PCP diagnoses that revealed HIV infection ranged from 23% to 55%, while none or few patients with a previously known HIV diagnosis were under cART [6]–[8].

In industrialized countries, fewer than 10% of patients taking cART experience virological failure [9]. In 2011, 82% of HIV-infected patients enrolled in the FHDH cohort had been on cART for at least 6 months, and 89% of them had a viral load (VL) below 50 copies/ml [10]. Therefore, the risk of presenting a PCP should be limited to basically late presenters. However, retention in care is an emerging problem: a meta-analysis conducted between 1995 and 2009 in the United States showed that only 59% of patients regularly attended medical appointments [11]. In 2013, some authors reported that half of the patients presenting with an ADI [12], [13] and specifically PCP [12], [13] were already in care. This raises concerns about long-term adherence to care and its repercussion on morbidity and mortality. Here we studied the characteristics and outcome of patients who presented a PCP diagnosis while already in care with or without a prior ADI and compared them with late presenter patients with an inaugural PCP during 2004–2011 in France. In particular the role of repetitive ADI on risk of mortality was investigated.

## Patients and Methods

### Patients

The French Hospital Database on HIV (FHDH) is a nationwide hospital-based cohort [14].This epidemiological network was created in 1989. Seventy participating hospitals across France provide data on HIV-infected patients, who represent 50–60% of all HIV-seropositive patients receiving care in France. The only FHDH inclusion criteria are HIV-1 or HIV-2 infection and written informed consent. Trained research assistants prospectively collect clinical, biological and therapeutic data and causes of death from medical records, using specialized software. For this analysis, we considered all HIV-1-infected patients enrolled in the FHDH who had a first diagnosis of PCP. PCP diagnosis was considered definitive if there was a histologic or cytologic evidence of *P. jirovecii* on bronchoalveolar lavage, sputum and/or lung biopsy specimens. Diagnosis was considered only presumptive if there was a recent history of dyspnea or nonproductive cough in the absence of evidence of bacterial pneumonia, and a positive clinical response to appropriate therapy for PCP. We excluded patients with no CD4 cell count within 3 months of PCP diagnosis and surviving patients with no CD4 cell count after PCP. Patients previously enrolled (PE) in the FHDH with an incident PCP were compared to patients with a PCP revealing HIV infection (taken as the reference group). The group of PE patients was divided into patients with a prior history of AIDS-defining illness (ADI) (PE prior ADI) and those without prior ADI (PE non prior ADI). ADI was defined as a C category according to the 1993 CDC criteria.

### Definition of adequate adherence to care

Adherence to care of patients enrolled in FHDH was considered adequate if they had a CD4 cell count at least every 6 months. The duration of adequate adherence to care was calculated as the sum of the number of days between two CD4 cell counts, after excluding days exceeding the time window of 6 months. For patients with no CD4 cell counts prior to PCP, the duration of adequate adherence to care were set at 0. The proportion of time spent with adequate adherence to care was determined by dividing the number of days with adequate adherence to care by the total length of follow up. This indicator of adherence to care was calculated for each patient with at least a visit in the period 2004–2011 and who were followed up for at least 3 years in the FHDH cohort. For PE with an incident PCP, adherence to care was first calculated from the first visit (enrolment in the FHDH) until PCP diagnosis; then adherence to care was calculated during the last 2 years before PCP. In the whole cohort, adherence to care was calculated from the first visit (enrolment in the FHDH) until the last one in the FHDH cohort.

### Statistical analysis

Incidence of PCP among PE patients was calculated for each year during the study period by dividing the number of PE PCP cases by the number of person-years followed during that year in the cohort. Frequency of inaugural PCP among newly enrolled patients was calculated for each year during the study period by dividing the number of PCP cases in the reference group by the number of new patients included in the cohort during the same year. Evolution of the proportion of PCP diagnosis in PE patients during the study period was compared by a Cochran Armitage test for trend.

Baseline characteristics were compared across the 3 groups, PE prior ADI, PE non-prior ADI and patients with a PCP revealing HIV infection (reference group) by using the χ2 test for categorical variables and the Kruskal-Wallis test for continuous variables. Kaplan-Meier estimates of the proportion of patients with virological control (HIV viral load [VL]≤50 copies/ml), the proportion of patients with immune reconstitution (CD4>200/mm^3^), and survival were compared between the three groups of patients after right censoring the data 3 years after PCP diagnosis.

Cox proportional hazards models were used to identify factors associated with outcome, including age (<30 years, ≥30 to <50, and ≥50), country of origin (sub-Saharan Africa, others), the HIV exposure category (men who have sex with men (MSM), intravenous drug users (IDU), heterosexual), CD4 cell counts at PCP diagnosis (CD4<50/mm^3^, 50≤CD4<100, 100≤CD4<200, CD4≥200), and HIV viral load at PCP diagnosis (VL<5 log copies/ml, VL≥5 log copies/ml). In the Cox model for immune reconstitution, virological control was included as time-dependent variable. In the Cox model for mortality, virological control and immune reconstitution were included as time-dependent variables.

All tests were two-sided, and P values below 0.05 were considered to denote statistical significance. All statistical analyses used the SAS software package (version 9.3; SAS Institute, Cary, NC, USA).

## Results

### Trends of PCP over time

**Figure 1:**
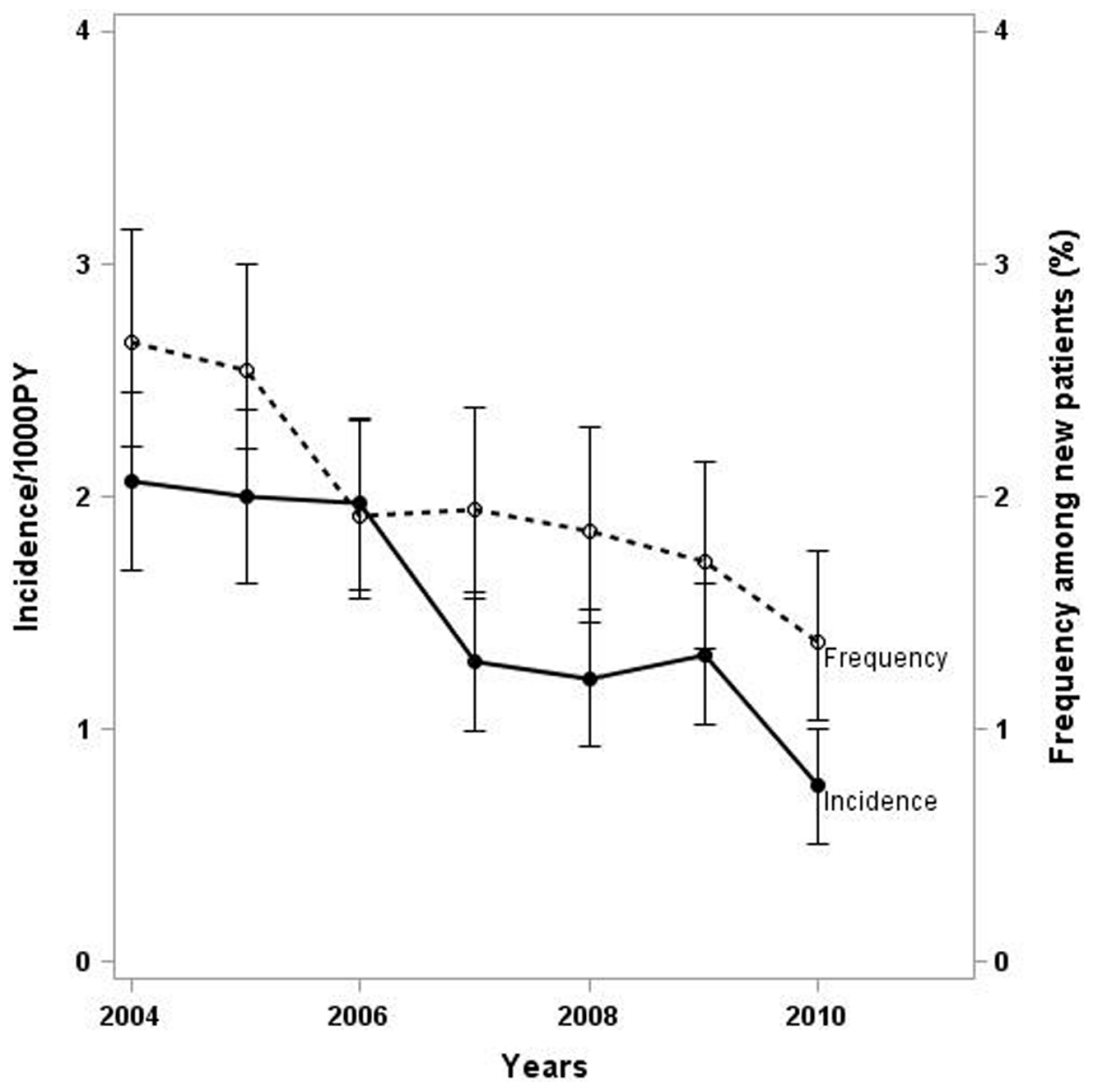
Trends in the risk of PCP overtime: incidence of first PCP for previously enrolled patients (bolt line) and frequency of inaugural PCP in newly enrolled patients (dotted line).

Over the period 2004–2011, 85,743 patients had a follow-up in the FHDH and 35,985 new patients were included in the FHDH cohort. There was a decreasing frequency of PCP in the group of patients with an inaugural PCP and a decreasing incidence of PCP for previously enrolled patients (figure 1). In addition, the proportion of PCP diagnosed in PE group among all PCP diagnosis has remained stable during the study period (46% in 2004 vs. 40% in 2010, Cochran Armitage test for trend p = 0.77).

### Characteristics of the patients

**Figure 2:**
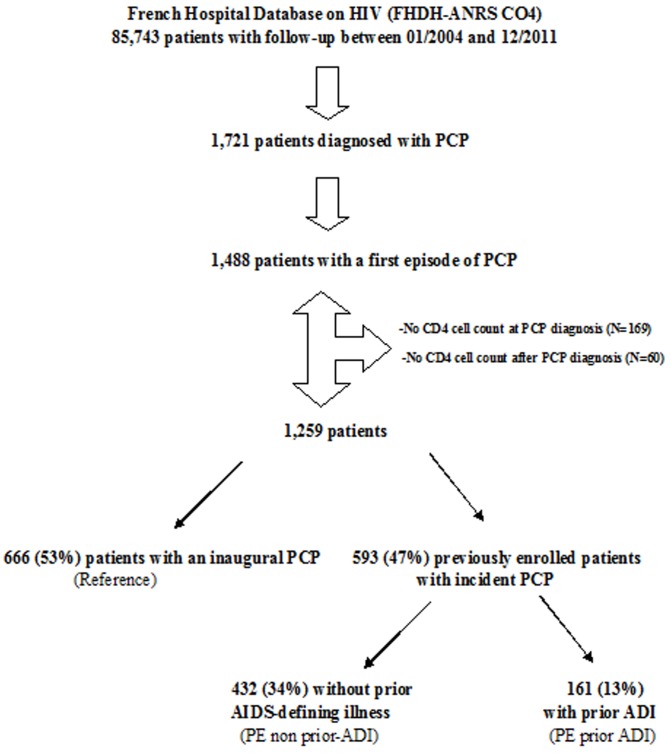
Patient selection flow diagram.

Over our study period a first episode of PCP was diagnosed in 1488 patients, of whom 1259 patients with at least a CD4 cell count within 3 months of PCP diagnosis and at least one CD4 cell count during follow-up were retained for analysis (figure 2).

The patients' characteristics at PCP diagnosis are shown in table 1. Overall, 593 (47%) patients were previously enrolled in the FHDH. The majority of patients were males (73%). Median age was 42 years (IQR, 36–49). Migrants represented 12% of the overall population, and sub-Saharan patients were more numerous among patients with a PCP revealing HIV infection. The IDU HIV transmission group was more numerous in the PE group. The median CD4 cell count and median viral load at PCP diagnosis were 38/mm^3^ (IQR, 15–92) and 5.2 log10 copies/ml (IQR, 4.6–5.7), respectively, with no difference of clinical importance between PE patients, and patients in the reference group.

**Table 1:** Patients with a first episode of PCP in 2004–2011: characteristics at PCP diagnosis.

	Previously Enrolled patients with an incident PCP	Previously Enrolled patients with an incident PCP without prior ADI	Previously Enrolled patients with an incident PCP with prior ADI	Patients with an inaugural PCP revealing HIV infection	P value°
	(all PE)	(PE non-prior ADI)	(PE prior ADI)	(reference)	
**Total**	593 (100%)	432 (100%)	161 (100%)	666 (100%)	
**Male gender**	417 (70%)	293 (68%)	124 (77%)	502 (75%)	0.001
**Age (years)**	42 (37–47)	42 (37–47)	42 (38–48)	42 (36–50)	0.55
**Sub-Saharan origin**	50 (8%)	32 (7%)	18 (11%)	103 (15%)	0.0003
**Transmission group**					<0.0001
MSM	154 (26%)	116 (27%)	38 (24%)	189 (28%)	
IDU	109 (18%)	68 (16%)	41 (25%)	20 (3%)	
Heterosexual	330 (56%)	248 (57%)	82 (51%)	457(69%)	
**CD4/mm3**	38 (15, 92)	53 (18,140)	33 (10,99)	34 (13,70)	<0.0001
**HIV viral load,** log10 copies/ml (N = 1169)	5.2 (4.6,5.7)	5.1 (4.1, 5.6)	5.0 (3.3, 5.6)	5.3 (4.8, 5.7)	<0.0001
**Time since enrolment** (years)	8.2 (3.7, 11.3)	8 (3.7,11.1)	8.7 (3.8,11.7)	NA	
**cART >1year**	400 (68%)	262 (61%)	138 (86%)	NA	
**Patients with cART >1 year**					
Duration of treatment (years)	7 (4.8–9)	6.7 (4.6, 8.8)	7.8 (5.1, 8.8)	NA	
CD4 nadir up to cART initiation	180 (81–300)	204 (106, 324)	119 (36, 220)	NA	
CD4>350/mm^3^ *	277 (69%)	191 (73%)	86 (62%)	NA	
HIV viral load <500 copies/ml **	318 (80%)	203 (77%)	115 (83%)	NA	

Figures are medians (Interquartile Range) or N (%); °P value: χ2 test for categorical variables and Kruskal-Wallis test for continuous variables, comparison between PE non-prior ADI, PE prior ADI, and reference groups.

* Percentage of patients with CD4 cell count >350/mm^3^ after cART initiation;

**percentage of patients with HIV viral load<500 copies/ml after cART initiation ADI: AIDS-defining illness, MSM: Men who have Sex with Men, IDU: Intravenous Drug User.

For PE patients, median time since enrolment was 8.2 years (IQR, 3.7–11.3) and 27% of them had had a prior ADI. Although 439 (74%) of these patients had already had a prescription for cART, all but 18 of them had detectable VL at PCP diagnosis (median, 5 log10 copies/ml [IQR, 4.1–5.6]). Among PE patients with at least one year of cART prescription before PCP, 277 (69%) patients have had at least once high CD4 cell count (CD4 cell count>350/mm^3^), and 318 (80%) patients a virological response (HIV viral load<500 copies/ml). Median proportion of time with adequate adherence to care was 60% (IQR, 33–84) since FHDH enrolment for PE patients until PCP diagnosis, and 45% (IQR, 1–81) during the 2 years before PCP diagnosis, compared to a median proportion of 85% (IQR, 66–96) for all FHDH enrollees.

### Virological, immunological and clinical outcomes

**Figure 3:**
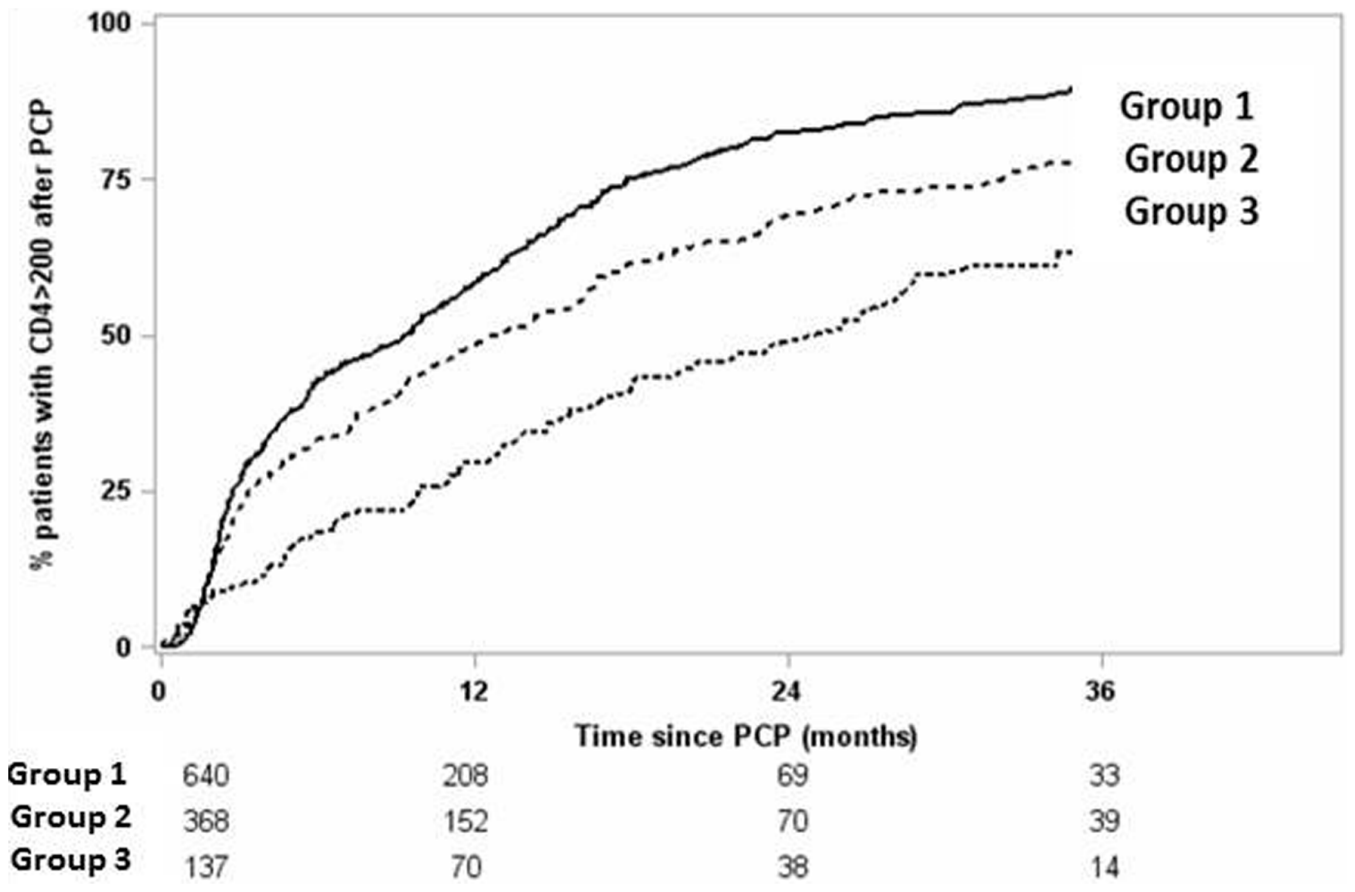
Kaplan-Meier estimates of immune reconstitution (CD4 cell count ≥200/mm^3^) probability, and number of patients at risk, after PCP diagnosis for patients with an inaugural PCP revealing HIV infection (reference, group 1), previously enrolled patients with incident PCP without prior AIDS-defining clinical illness (ADI) (PE non-prior ADI, group 2) and previously enrolled patients with incident PCP with prior ADI (PE prior ADI, group3).

One year after PCP diagnosis, VL was ≤50 copies/ml in 64% of PE non-prior ADI patients, 49% of PE prior ADI patients and 76% of patients in the reference group (p<0.0001). Among the 1145 patients (91%) with CD4 cell counts below 200/mm^3^ at PCP diagnosis, 52% had CD4 cell counts ≥200/mm^3^ one year after PCP diagnosis, and 84% at three years. At 1 and 3 years, respectively, CD4 cell counts ≥200/mm^3^ were achieved in 48% and 78% of PE non- prior ADI patients, and 29% and 68% of PE prior ADI patients and 58% and 90% of patients in the reference group (figure 3). PE non-prior ADI patients (HR, 0.7 [95%CI, 0.6–0.8]) and PE prior ADI patients (HR, 0.6 [95%CI, 0.4–0.8]) had slower immune reconstitution than patients in the reference group (table 2).

**Table 2:** Factors associated with immune reconstitution (CD4>200/mm^3^) within 3 years after PCP.

	Total	Nb of events within 3 years after PCP	Univariate analysis	Adjusted analysis	P value
			HR* (95%CI)	HR* (95%CI)	
**Patient group**					<0.0001
Reference group with inaugural PCP	640	468	1.0	1.0	
PE non-prior ADI patients	368	236	0.7 [0.6–0.8]	0.7 [0.6–0.9]	
PE prior ADI patients	137	63	0.4 [0.3–0.6]	0.6 [0.4–0.8]	
**Age (years]**					0.62
<30	93	60	1.0 [0.8–1.3]	1.1 [0.8–1.4]	
[30–49]	802	540	1	1	
≥50	250	167	1.2[1.0–1.4]	1.1 [0.9–1.3]	
**Transmission group**					0.001
MSM	308	230	1.3 [1.2–1.6]	1.1 [0.9–1.3]	
Heterosexual	724	481	1	1	
IDU	113	56	0.6 [0.5–0.8]	0.6 [0.5–0.8]	
**Origin**					0.0002
Sub-Saharan	146	78	1	1	
Other	999	689	1.7 [1.3–2.1]	1.6 [1.2–2.1]	
**CD4 at PCP diagnosis (/mm^3^]**					<0.0001
[0–49]	718	439	1	1	
[50–99]	245	181	2.0 [1.7–2.4]	2.2 [1.8–2.6]	
[100–199]	182	767	2.6 [2.1–3.1]	3.1 [2.6–3.9]	
**HIV viral load (VL) at PCP diagnosis**					<0.0001
<5 log10 copies/ml	405	256	1	1	
≥5 log10 copies/ml	662	468	1.3 [1.1–1.5]	1.4 [1.2–1.7]	
**HIV VL≤50 copies/ml after PCP (time-dependent covariable)**					<0.0001
No	NA	NA	1	1	
Yes	NA	NA	2. 3 [1.9–2.9]	2.3 [1.8–2.8]	

*Adjusted HR>1: in favor of achieving a CD4 cell count ≥200/mm^3^; PE non prior ADI: previously enrolled patients without prior AIDS-defining illness, PE prior ADI: previously enrolled patients with prior AIDS-defining illness, MSM: Men who have Sex with Men, IDU: Intravenous Drug User.

Mortality at 1, and 3, years after PCP diagnosis was 7%, and 11%, respectively. In subgroup analyses, mortality at 1 and 3 years was respectively 5% and 8% among PE non-prior ADI patients, 16% and 25% among PE prior ADI patients and 6% and 9% among patients in the reference group (p<0.0001). After adjustment on baseline characteristics, PE prior ADI patients were at increased risk of mortality (HR, 2.7 [95%CI, 1.7–4.2]) compared to patients in the reference group (Table 3). This higher mortality risk for PE prior ADI patients remained after adjustment on viral suppression and immune reconstitution (HR, 2.4 [95%CI, 1.5–3.7]).

**Table 3:** Factors associated with risk of death after PCP within 3 years after PCP.

	Total	Nb of events within 3 years after PCP	Univariate analysis	Model I: Adjusted analysis	P value	Model II: Adjusted analysis	P value
			HR* (95%CI)	HR* (95%CI)		HR* (95%CI)	
**Patient group**					<0.0001		<0.0001
Reference group with inaugural PCP	666	52	1	1		1	
PE non-prior ADI patients	432	29	0.8 [0.5–1.3]	0.8 [0.5–1.3]		0.8 [0.5–1.3]	
PE prior ADI patients	161	35	2.9 [1.9–4.4]	2.7 [1.7–4.2]		2.4 [1.5–3.7]	
**Age (years]**					0.002		0.0009
<30	98	9	1.3 [0.6–2.6]	1.4 [0.7–2.9]		1.3 [0.6–2.7]	
[30–49]	888	68	1	1		1	
≥50	273	39	2.0 [1.3–2.9]	2.1 [1.4–3.1]		2.2 [1.4–3.3]	
**Transmission group**					0.08		0.12
MSM	343	24	0.8 [0.5–1.2]	0. 8 [0.5–1.2]		0. 8 [0.5–1.3]	
Heterosexual	787	72	1	1		1	
IDU	129	20	1.7 [1.1–2.8]	1.6 [0.9–2.7]		1.5 [0.9–2.6]	
**Origin**					0.45		0.24
Sub-Saharan	153	12	1	1		1	
Other	1106	104	1.2 [0.6–2.1]	1.3 [0.7–2.4]		1.5 [0.8–2.7]	
**HIV VL≤50 copies/ml after PCP (time-dependent covariable)**							0.0009
No	NA	NA	1			1	
Yes	NA	NA	0.3 [0.2–0.6]			0.4 [0.2–0.7]	
**CD4>200/mm^3^ after PCP (time-dependent covariable)**							0.0008
No	NA	NA	1			1	
Yes	NA	NA	0.3 [0.2–0.5]			0.4 [0.3–0.7]	

*Adjusted HR>1: in favor of an increased risk of death; PE non prior ADI: previously enrolled patients without prior AIDS-defining illness, PE prior ADI: previously enrolled patients with prior AIDS-defining illness, MSM: Men who have Sex with Men, IDU: Intravenous Drug User.

## Discussion

In a context of a decreasing risk of PCP, 47% of patients diagnosed with PCP have already been in care but presented a poor adherence to care during the 2 years prior to PCP diagnosis. In addition, the 3 year mortality rate after PCP diagnosis was very high (25%) for previously enrolled patients with prior ADI, higher than in previously enrolled patients without prior ADI and the reference group of patients with an inaugural PCP revealing HIV infection.

This is the largest observational study of PCP in the recent cART era; however several limitations must be discussed. First, we used PCP diagnoses and deaths reported in the FHDH cohort. A previous analysis of this cohort showed that 93% of PCP diagnoses were confirmed on examining the patients' clinical records [15]. Second, among 1488 patients with a first episode of PCP during the study period, 229 patients were excluded from this analysis because of a lack of CD4 cell counts at PCP diagnosis or during subsequent follow-up. No excess mortality was observed in this patient subgroup (data not shown). Third, the rate of loss to follow up at 3 years after PCP was 16%. But owing to the French law that prevents nominative or indirectly nominative data collection in FHDH; vital status could not be checked against the national death registry. However no difference in rate of loss of follow-up was observed between the 3 groups (Log Rank test p = 0.74).

In previous studies of PCP few patients had been prescribed cART before PCP onset [6]–[8] but an increase of the proportion of ADI and specifically of PCP have been recently reported in pretreated HIV infected patients followed up in industrialized countries despite free access to care [12], [13]. In our study, among previously enrolled patients, 2/3 had had at least a cART prescription for more than one year before PCP. Before PCP, 67% of patients with at least one year of cART prescription have had an immunological response and 80% a virological response; theoretically those patients should not have presented a PCP episode. Since all PE patients had very low CD4 cell count and high VL at PCP diagnosis, it could be hypothesized that for some patients, adherence had diminished over time with a risk of developing ADI. Patients are now facing the challenge of life-long adherence to medications and care and our policy must evolve. Efforts have been focused on adherence at the beginning of cART. We need now to concentrate on patients followed up for many years and design interventions to lower the risk of decreasing adherence over time.

It was striking to note that PE patients (with and without prior ADI) had a worse immune reconstitution than the reference group. CD4-nadir before cART initiation has been shown as the strongest predictor of the immune response [16], [17]. The extent of prior CD4 T cell depletion determined the size of the viral reservoir after prolonged suppressive antiretroviral therapy [18]. In our study, PE prior ADI patients have had a lower CD4 nadir at cART initiation (median of CD4: 119/mm^3^ (IQR, 36–220)) compared to PE non-prior ADI patients, (median of CD4: 204/mm^3^ (IQR, 106–324)). This could explain the difference of immune reconstitution after PCP between these two groups of patients. The role of antiretroviral drug resistance could also be hypothesized to explain that previously enrolled patients had a lower rate of immune reconstitution than patients with PCP revealing HIV. The time required to reach a CD4 cell count above 300/mm^3^ has been shown to increase with the number of failed regimens [19].

Despite cART, having an ADI like PCP is still associated with a poor vital prognosis. Overall, in the last fifteen years, the 3-year post-PCP mortality rate have not changed in the FHDH cohort from 12% in 1998–2000 [1] to 11% in the present study. However, a special group of patients with very high mortality (25%, PE prior ADI patients), was identified in our study, probably explaining why the overall mortality rate has not fallen. Since IDU were more numerous in PE prior ADI patients, a sensitivity analysis was undertaken in order to see if part of the poor prognosis could be explained by non-AIDS cause of death such as overdose. In this sensitivity analysis excluding the IDU group, an increase of risk of death was still observed for PE prior ADI patients (HR = 2.6 [1.6–4.3]).

Patients in the PE prior ADI group who presented with another ADI, here PCP, had a poorer prognosis that persisted after adjusting on baseline characteristics and on immune reconstitution and viral suppression. HIV viral load and CD4 cell counts are strong predictors of the risk of death but having repeated ADI is contributing to the risk of death beyond its impact on immune reconstitution and viral suppression. This is in line with the results of a large collaborative European study that showed a persistent role of prior AIDS on the risk of death, even in patients with CD4 cell counts ≥500/mm^3^ [20]. One hypothesis may be an immune system dysfunction not exclusively translated in the CD4 cell count which could deepen at each ADI. Clinicians must be aware of the pejorative effect of an ADI even at a time where powerful treatments are available. Warning message on importance of maintaining viral suppression and immune reconstitution to prevent any new ADI must be regularly reinforced and especially for patients with a previous ADI.

## Conclusion

In a context of diminishing risk of PCP, half of the patients diagnosed with PCP in the recent cART period were already in care; the majority of them had already started cART but with vanishing adherence to care. Efforts need to be stressed on those patients with a history of previous ADI in order to prevent further event which will increase mortality. The consequences of having had an ADI need to be explained to patients as well as the necessity of maintaining an excellent observance and never stop cART.
